# How to Manage Impacted Third Molars: Germectomy or Delayed Removal? A Systematic Literature Review

**DOI:** 10.3390/medicina55030079

**Published:** 2019-03-26

**Authors:** Edoardo Staderini, Romeo Patini, Federica Guglielmi, Andrea Camodeca, Patrizia Gallenzi

**Affiliations:** Fondazione Policlinico Universitario A. Gemelli IRCCS, Institute of Dentistry and Maxillofacial Surgery, Università Cattolica del Sacro Cuore, 00168 Rome, Italy; edoardo.staderini@yahoo.it (E.S.); fe.guglielmi@gmail.com (F.G.); andrecamo@tiscali.it (A.C.); patrizia.gallenzi@unicatt.it (P.G.)

**Keywords:** third molar germs, orthodontics, oral surgery, dental impaction, complications, systematic review

## Abstract

*Objectives:* The aim of this systematic review is to evaluate and compare the risks and benefits of germectomy and delayed removal of third molars and develop a patient management algorithm for second molar eruption in syndrome/incompliant patients. *Materials and Methods:* A literature search was performed in the following databases; the Cochrane Central Register of Controlled Trials (CENTRAL), PubMed, Scopus, and Web of Science. Last search was done on July 2nd, 2018 including articles published from the last 18 years. The search aimed to identify all relevant studies written in English language. Gray literature was excluded. Risk of bias was evaluated with specific predetermined criteria. This systematic literature review was reported according to the PRISMA-P statement and was registered in the PROSPERO database with the following protocol ID: 104261. *Results*: Literature search war performed on July 2018 and updated on February 2019. A total of 1610 articles were screened. After abstract screening and discarding duplicates, 86 full-text articles were obtained and subjected to additional evaluation. Four articles were included in the review. Three studies were considered as having a medium risk of bias and one was assessed as at high risk. Due to the heterogeneity of presenting results and a very low number of included studies a quantitative analysis was not possible. Only qualitative analysis was made. Considering the limited number of studies included and the level of risk of bias there is no sufficient evidence to state the benefits of preventive removal of impacted third molars, especially in patients with poor oral hygiene due to intellectual disability. Early germectomy represents an elective approach of pathologic alteration of tooth germ; orthodontic issues meet appropriate indication for a delayed removal. *Conclusions*: Given the best evidence-based information regarding patients’ medical condition, we highlight the need to provide an ethical-based comprehensive approach in the diagnostic workflow and the assessment of treatment outcome.

## 1. Introduction

Tooth impaction is a pathological situation where a tooth fails to attain its normal functional position. A relationship between the presence and the role of the mandibular third molar as a physical obstacle of the eruption path of the mandibular second molar has been hypothesized, even if the most recent evidences seem to confirm the results of Kaplan, who suggested that the impaction of the mandibular second molar can be related primarily to an arch length deficiency [1,2]. When absence of the second molar is observed at least two years behind is scheduled time, a radiographic examination is required to disclose if the cessation of eruption of the second molar is associated to an abnormal inclination of the wisdom teeth [3,4]. There are, however, conflicting opinions about the need or not to extract the gem of the third molar to facilitate the alignment of the second molar as not all authors believe that this germ can be an obstacle to the eruptive path of the second molar [5].

Among the reasons why some clinicians do not consider the preventive extraction of the germ of the third mandibular molars there is the possibility of postoperative complications. Furthermore, an additional source of debate is represented by the evidence that third molar germectomy seems to be connected to less morbidity than extraction of third molars with formed roots. So, some authors underline the necessity of carrying out a very careful assessing of cost–benefit ratio for evaluating the indications of third molar early or delayed extraction [6].

Unfortunately, incompliant/syndrome patients often require general anesthesia to remove wisdom teeth; dental practitioners and oral surgeons have a wide variation of judgments regarding the indication of early or delayed extraction of third molar gems [7,8].

This lack of global consensus on all the aspects described above leads to the concept that early extraction of the third molar gems is decided on an empirical basis.

The aim of this systematic review is to evaluate the rate of surgical complications associated to germectomy (including the general anesthesia for syndrome/incompliant patients) and the benefits in terms of duration of therapy for correction of the second molar inclination after the removal of third molar germs.

## 2. Materials and Methods

The Preferred Reporting Items for Systematic Review and Meta-Analyses (PRISMA) checklist (Figure S1) was used as a guideline for conducting and reporting the present systematic review [9]. The protocol of the present review was registered in the PROSPERO database with protocol ID 104261.

### 2.1. Review Question

The following focused question, formulated in the Patient, Intervention, Comparison and Outcome (PICO) format was developed: “Does early extraction of third molar germs produce a benefit in terms of duration of therapy for correction of second molar inclination or in terms of surgical complications? Is it possible to draw some recommendations from the literature reports, especially for syndrome/incompliant patients?”

### 2.2. Selection Criteria

#### 2.2.1. Participants

Studies were conducted on participants who were suggested to extract third molar germs at the age of 18 years or under.

#### 2.2.2. Interventions

Any intervention or combination of surgical techniques given for the extraction of third molar germs, including early or delayed germectomy.

#### 2.2.3. Outcomes

Any clinical outcome, including (but not restricted to) complications of germectomy and duration of therapy for correction of second molar inclination between treated and untreated subjects.

#### 2.2.4. Study Design

Studies were included if they were case–control or cohort study with prospective or retrospective design or if they dealt with recommendations regarding the management of third molar germs. Studies were selected if they were published in the last 18 years in English, French, German, Spanish, or Italian.

Excluded articles:

- Publications in languages other than English, French, German, Spanish, or Italian.
- Case reports, case series, review articles, abstracts, and discussions.
- Studies that evaluated less than five patients.

### 2.3. Information Sources and Search

The literature search war performed on July 2018 and updated on February 2019. The following electronic databases were systematically searched; MEDLINE-PubMed and all evidence-based medicine reviews via Web of Science, Scopus, and the Cochrane Central Register of Controlled Trials (CENTRAL).

The combination of MeSH terms and free text words used for MEDLINE-Pubmed database are as follows.

#1 (Molar, Third/) OR ((“third molar *” OR “wisdom tooth” OR “wisdom teeth” OR “3rd molar *” OR third-molar).mp.)
#2 (((Tooth, impacted/) OR ((tooth impact *) OR (teeth impact *).mp.)) OR Tooth, unerupted/)
#3 third molar * germ OR wisdom tooth germ OR wisdom teeth germ OR 3rd molar* germ OR third.molar germ.mp.
#4 #1 AND (#2 OR #3)
#5 (((Tooth extraction/indications) OR ((extract * OR remov *)/indications)) OR Tooth extraction/early) OR Tooth extraction/early
#6 #4 AND #5
#7 third molar * germectomy OR wisdom tooth germectomy OR wisdom teeth germectomy OR 3rd molar * germectomy OR third-molar germectomy
#8 #6 OR #7.

The MeSH terms “Syndromes” or “Patient Compliance”, or other variations related to the keywords, were not included in order to make the list of searches more concise; therefore, we included those search terms in the selection criteria to focus the search results.

This search strategy was firstly designed for Medline and then adapted for the other databases.

A supplementary manual search was performed of the following peer-reviewed journals for articles published between January 2000 and December 2018: European Journal of Orthodontics, American Journal of Orthodontics and Dentofacial Orthopedics, Angle Orthodontist, Progress in Orthodontics, International Journal of Pediatric Dentistry, Journal of Oral & Maxillofacial Surgery, International Journal of Oral and Maxillofacial Surgery, British Journal of Oral & Maxillofacial Surgery, and Journal of Cranio-maxillofacial Surgery. In addition, the bibliographies of all selected articles were checked and all corresponding authors of included articles were contacted by e-mail in order to recover unpublished articles or raw data and to include as many relevant studies as possible in the analysis.

### 2.4. Study Selection

Screening process was conducted independently and in duplicate, two reviewers (FG and AC) evaluated the titles and abstracts of the retrieved studies from the database searches using the inclusion criteria. Subsequently, the same reviewers performed the assessment of the full-text articles. Any disagreements were solved through discussion until consensus.

### 2.5. Data Collection Process

The data were first extracted independently and in duplicate by the same reviewers (FG and AC) using specially designed data extraction forms. For studies matching with the inclusion criteria, or for which information in the title and abstract was insufficient to make a clear decision, the reviewers obtained and screened the full report.

### 2.6. Data Items

The variables extracted from each selected article included: study type, sample size, population details (including age and gender), complications, and follow-up.

### 2.7. Outcomes

The outcomes were the complications rate of germectomy as well as the benefit in terms of duration of therapy for correction of second molar inclination between treated and untreated subjects and the factors associated with the recommendations to extract third molar germs.

### 2.8. Risk of Bias in Individual Studies and Quality of Evidence

The reviewers independently evaluated the outcomes through a structured form.

Methodological quality scores were given according to predetermined custom-built criteria and the quality of evidence was assessed as high, moderate, or low for each study.

## 3. Results

The initial electronic search resulted in a total of 1610 titles (Databases: MEDLINE-Pubmed, Scopus, CENTRAL, and Web of Science). After the independent elimination of duplicate articles, a total of 1379 titles were considered for possible inclusion. A total of 1293 articles were removed based on their title and abstract; therefore, 86 full-text articles were selected. Among these studies, four were included in the review [2,10,11,12]. After accurate checking of the bibliographies of all selected articles no additional publications were recovered.

**Figure 1:**
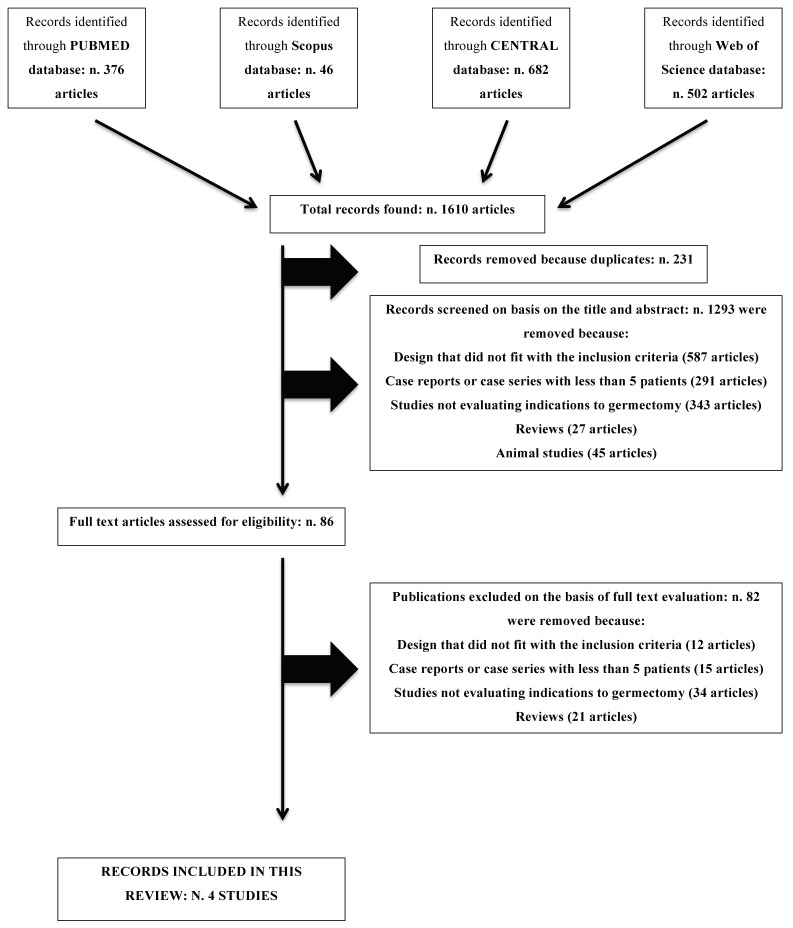
Preferred Reporting Items for Systematic Review and Meta-Analyses (PRISMA) flow diagram for the identification and selection of studies.

In conclusion, only four studies were identified as potentially eligible for inclusion in this review (Figure 1).

### 3.1. Exclusion of Studies

After full-text evaluation, twelve articles were excluded because their design did not fit the inclusion criteria. Fifteen studies were excluded because they were case reports or case series with less than five patients treated, thirty-four studies were excluded because they did not evaluate indications to germectomy procedure, and twenty-one studies were excluded because they were only narrative reviews about the topic.

### 3.2. Included Studies

Two studies were carried out in USA [11,12], one in Italy [2], and one in Spain [10].

Three studies were conducted at University dental clinics [2,10,12] and one study enrolled patients coming from private dental centers [11]. Except for one study that could be considered a position paper regarding the USA dental school departments’ recommendations about early third molar extraction [12], the Spanish study was conducted according to a retrospective design [10], while the other two were prospective [2,11].

Characteristics of all the included studies are summarized in Table 1.

Overall the included studies were very heterogeneous because they did not always report data regarding the outcomes of this systematic review. Furthermore, in some cases authors presented the data graphically preventing the authors of this article from retrieving them accurately. For these reasons, a quantitative analysis of the results was not possible; only a qualitative analysis was made.

### 3.3. Characteristics of Participants

The selected studies included adolescents (age range: 12–18 years) and adult patients (older than 21 years) candidate or subjected to third molar surgical extraction according to a retrospective or prospective design; one study reported data drawn from questionnaires mailed to department chairpersons regarding recommendations about early third molar extraction [12]. In accordance with the aims of the present review, in articles that reported data about populations of adolescents and adults, only data regarding patients younger than 18 years old were collected.

Patients were excluded if they matched at least one of the following exclusion criteria: active caries lesions, orthodontic appliances, previous extraction of one third molar, and any systemic disease or syndrome.

### 3.4. Characteristics of Interventions

Data regarding the sample site, population details, eventual complications, and follow-up periods are summarized in Table 1. Studies were conducted in the last 13 years. A total of 984 individuals candidate to third molar extraction and 146 reports from questionnaires filled by department chairpersons were evaluated. Among individuals, 727 were younger than 18 years old, and were considered in this analysis. Only one study reported information regarding surgical complications after third molar germs extraction [10]. The follow-up period was not homogeneous and always shorter than 20 months.

### 3.5. Characteristics of Outcome Measures

Only one article evaluates the rate of surgical complications associated to germectomy [10], and another one reported the benefit of third molar germectomy in terms of duration of therapy for correction of second molar inclination [2]. The overview of dental school departments’ and general dentists’ recommendations regarding the topic was reported in two of the included articles [11,12].

### 3.6. Risk of Bias in Included Studies and Strength of Evidence

The risk of bias is summarized in Table 2. According to a predefined custom-built checklist, one study was assessed as at high risk [12], and three studies were assessed as at medium risk [2,10,11].

### 3.7. Effects of Interventions

#### 3.7.1. Surgical Complications

Chaparro-Avendaño et al. analyzed the incidence of complications following third molar surgical extraction in patients between 12 and 18 years of age, with an evaluation of the association between such complications and patient age and sex, the reason for extraction, the degree of dental development, and third molar position, angle, and impaction [10]. The postextraction complication rate was 15.62%, and all complications were mild and reversible, comprised persistent swelling and pain (8.9%), secondary infection (1.8%), difficulty in opening the mouth (2.3%), and ecchymosis (2.1%). One case each of inferior alveolar nerve paresthesia (0.26%) and lingual nerve paresthesia (0.26%) was recorded in Group C (17–18 years of age). The overall incidence of complications was not significant even if they were significantly more frequent in females (18%) than in males (9.3%) (*p* = 0.033). No statistically significant differences were found regarding the association between complications and the third molar calcification stage or the degree of impaction in upper third molars. This contrasted with the situation in the case of the lower third molars, where the teeth presenting impaction Classes III, II, and I accounted for 27.8%, 17.6% and 3.7% of the complications, respectively (*p* = 0.028). In relation to angulation, distally angled molars posed most complications (41.7%), followed by mesially inclined teeth (19.1%) and vertical molars (9.5%) (*p* = 0.048). Regarding the reasons for extraction, the group of patients in whom extraction was indicated for early reasons accounted for 11.7% of the complications, while extraction due to clinical symptoms and orthodontic reasons accounted for 17.9% and 18.4% of the complications, respectively. Nevertheless, no statistically significant differences were observed between the reasons for extraction and the incidence of postoperative complications (*p* = 0.221).

#### 3.7.2. Clinical Recommendations

The position paper regarding recommendations of the US dental school departments published by Jasinevicius et al. in 2008 confirmed the same recommendations derived from the previous department chairpersons’ questionnaires results of 1998/99 [12]. Over 75% of US departmental chairpersons of Oral Surgery, Orthodontics, and Restorative Dentistry divisions recommended early removal of third molar germs in adolescents. The article also investigated the rationale for early germectomy of third molars and identified such motivations: risk of future pathology/infections (including pericoronitis and caries) associated with third molars (75% of respondents), insufficient space (72% of respondents), and lack of functionality (27% of respondents).

The cohort study designed by Cunha-Cruz et al. gave information regarding recommendations for third molar removal among private practice dentists belonging to the network named PRECEDENT (Northwest Practice-based REsearch Collaborative in Evidence-based DENTistry) [11]. Dentists belonging to this network gave recommendations to third molar early extraction to prevent future problems (79%) or in case of unfavorable orientation according to their opinion (57%); only a limited number of dentists suggested early extraction in order to avoid the risk of future pathologies like: pericoronitis (4%), dental caries (4%), or other unspecified pathologies (1%).

#### 3.7.3. Correction of Second Molar Inclination

Cassetta and Altieri designed a prospective pilot study in which they evaluated the benefit of germectomy in terms of duration of therapy for the correction of second molar inclination respect to the luxation and positioning of a brass wire [2]. The second molar impaction was corrected in all patients, but there was no statistically significant difference in treatment time between the two groups (*p* = 0.440).

## 4. Discussion

The analysis of the results of the studies published in the last 18 years and included in this systematic review has revealed that there is no evidence to sustain the hypothesis that the third molar germectomy can reduce the treatment time for the correction of second molar inclination. This result gives more strength to the evidence already present in the literature that states that the correction of ectopic eruption of the permanent molars can be achieved even utilizing the brass wire technique alone [13]. Despite the previous evidence has already been extensively described, to the best of our knowledge it is already unclear under what circumstances the third molar germs should be extracted or not for the resolution of the impaction of the second molar. However, the aspect on which many authors agree is that it is advisable to abstain from the early extraction of the third molar as this intervention can expose the patient to a greater risk of postoperative complication [6,14,15].

On the one hand, scientific literature has already required for many years clinicians a decision-making approach strongly based on the cost–benefit regarding the early extraction of third molars [6]. In fact, according to the NIH 1989 Consensus Development Conference, [16] the third molar surgery should be performed only in case of

- morphostructural alterations or ectopic impactions;
- eruption not allowed by dysplastic alterations of the tooth germ or pathologic processes of the bone;
- necessity of distalization of first or second molar; or
- excessive anteroposterior growth or severe dentoalveolar discrepancy.

On the other hand, controversy exists over the extraction of third molars which have caused no pathology at all and this element has been extensively studied by some authors with the aim of defining the indication to germectomy [17,18,19].

Regarding the risk of postoperative complications following germectomy, this systematic review confirms the evidences already found in the previous reports: female patients are more susceptible to develop postoperative complications, and distally angled third molars or molars presenting Class III impaction are more prone to cause such complications [20,21]. No other type of complication has been found to be significantly more prevalent in early when compared to delayed removal of impacted third molars. One important advantage of germectomy that clinicians should take into account is that such type of intervention is less likely to cause inferior alveolar or lingual nerve damage as the roots of the molar have not yet been fully formed [22,23,24]. Therefore, there is almost a nonexistent relation of a tooth germ to the above-mentioned nerves.

According to the evidences found in the present review, the dental school departments’ and general dentists’ recommendations regarding the early removal of third molar germs remain high both in the presence and in the absence of pathology associated with the tooth. Because of the general lack of strong evidences from a methodological point of view regarding this topic it is believed that the decisions taken by the clinicians are often based on personal values and habitual practices [25,26,27,28]. The watchful waiting strategy based on several studies that noted that a percentage of impacted third molars completely righted over a period of time seems to be more common in private dentists [29,30,31] than in university clinics [32,33].

An important limitation of this review is that all results were drawn from only four studies due to the lack of reports that matched with the inclusion criteria. Moreover, some studies included patients belonging to a wide range of age and divided them by age groups. The authors of the present review included results taken from the group closest to the age in which the third molar is present at the stage of germ; this assumption could not be valid in all cases.

Only studies conducted in the USA reported recommendations of the dental school departments and general dentists about the early removal of third molar germs, for this reason the indications derived from those reports should not be considered as shared all over the world.

Lastly, the general risk of bias judged as medium/high imposes a very cautious interpretation of the results.

### 4.1. Clinical Recommendations for Syndrome/Incompliant Patients

Lower third molar extraction in syndrome/incompliant patients is a case-based therapeutic treatment; clinical approach is aimed to maximize risk–benefit ratio, basing on four considerations: risk of nonintervention, risk of intervention, benefit of nonintervention and benefit of intervention (Table 3).

### 4.2. Risk of Nonintervention

Toothache may trigger organic self-mutilation. Syndromes, mental retardation, and autism are often associated with organic self-injuries, defined as unconscious compulsive mutilation behavior not related to specific intent. Self-injuries may involve perioral region (tooth grinding, biting lower lip, tongue, and cheeks) or other anatomical districts (traumatic injuries on head, neck, and hands).

Consequences—wounds pain and tooth sensitivity—provoked by self-mutilation might induce themselves the onset of a negative loop characterized by persistence or even increase of the compulsive self-injuring behavior.

Moreover, the removal of the environmental clue is highly recommended in patients with Lesch–Nyhan syndrome, a heterogeneous group of neurological disorders, congenital insensitivity to pain with anhidrosis, and mental retardation.

Some antiepileptic drugs (i.e., phenytoin) may induce gingival hypertrophy, thus increasing the risk of periodontal complications of lower third molars. Among patients with recurrent epilepsy, the pain elicited by toothache makes the patient uncomfortable and triggers the onset of seizures [34].

### 4.3. Risk of Intervention

Surgical technique for germectomies is highly predictable; as above mentioned, tooth extraction is associated with mild and reversible surgical complications if root development is not completed. Swelling, discomfort, and reduced mouth opening are common sequels that gradually resolve over the next two weeks. Lower incidence of morbidity means less economic hardships from time off work for patient and caregivers [32].

Anesthesiologic side effects are often associated with the drug administration and the difficulty in safely managing syndrome patients (endotracheal intubation) [35]; however, the duration of surgical removal of tooth germs is lower when compared to the extraction of fully developed teeth, thus reducing the dose of administered anesthesia.

### 4.4. Benefit of Nonintervention

Avoidance of risk related to anesthesiologic procedures; syndrome or incompliant patients are eligible for third molar removal under general anesthesia. Respect to regional anesthesia, general anesthesia allows treating incompliant or phobic patients and extract multiple teeth. However, if syndrome/incompliant subjects are meeting criteria for tooth extraction and need to be treated in general anesthesia for many reasons, they should undergo all the procedures at the same time [36].

Furthermore, before planning a germectomy, it is also mandatory to evaluate the absence of pathologies of the young age that may cause premature loss of dental elements [37]. In patients with mental disability and neglected oral hygiene, wisdom teeth may substitute second molars, extracted for periodontal/restorative problems, and compensate loss of an important tooth as a retention element for fixed or removable prosthetic rehabilitations [38].

### 4.5. Benefit of Intervention

Under general anesthesia it is possible to remove third molars altogether. Based on our experience, the concomitant removal of all the third molars is needful in patients with a higher incidence of airway management problems; patients who had multiple anesthesiologic procedures or an ASA score of III need a careful examination of the reasons for the extraction of the lower third molars and, once required, they must solve all the dental issues in the same intervention.

On the other hand, a longer surgical procedure implies a higher dose of anesthesia and is more often associated with delayed onset infections on mandibular third molar sites. However, to avoid these complications, methods of drug premedication and telephone monitoring of patients have recently been proposed [39,40].

### 4.6. Indications for Third Molar Extraction in Syndrome Patients—Early Removal

We set the necessity of undergoing an intervention in general anesthesia as “minimum requirement” to hypothesize the removal of third molars in incompliant/syndrome patients.

Early removal meets appropriate indication in case of impaction, delayed or ectopic eruption of the neighboring teeth and in pathological situations such as follicular cyst formation.

Lower third molars presenting partial soft tissue impaction have a relatively hopeless long-term prognosis, because the lack of ability in performing daily oral health care may result in severe pericoronitis and extensive decay.

Furthermore, mesially impacted lower third molars might elicit grossly decay, frequent periodontal problems, and root resorption on the distal aspect of the second molars.

Cellulitis, abscess, temporary local swelling, pain, and mouth opening require prompt intervention as evidences of pathology.

### 4.7. Indications for Third Molar Extraction in Syndrome Patients—Late Removal

**Figure 2:**
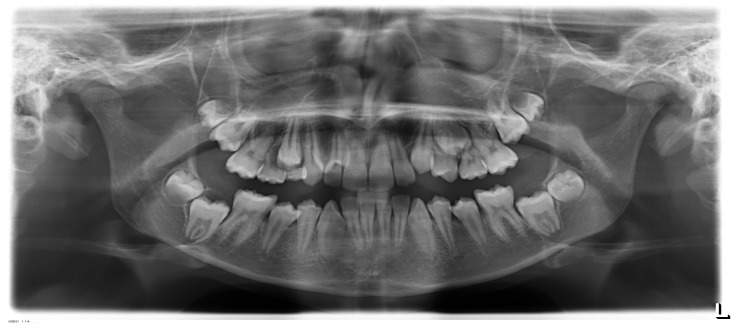
Lower third molars associated with a delay of eruption of second molars in a 12-year-old patient.

Spatial limitations represent a major indication for third molar extraction only if tooth interferes with the eruption of the adjacent teeth (Figure 2); crowding and displacement of permanent teeth may be superseded, as syndrome patients’ multiple loss of teeth due to poor oral hygiene provides the space required for successful correction of tooth misalignment. Collaboration between the orthodontist and the surgeon provides a multidisciplinary diagnosis and treatment plan to be generated.

## 5. Conclusions

Considering the limited number of studies included and the level of risk of bias there is no sufficient evidence to define absolute indications or contraindications for preventive removal of impacted third molars. Pros and cons of such intervention must be verified by further studies that are encouraged to be conducted with a prospective design, following appropriate scientific guidelines.

In agreement with NICE (National Institute for Health and Care Excellence) guidelines, third molars cannot be considered “functionless teeth”, especially in patients with mental disability, when they often replace missing teeth.

First of the NICE guidelines is “the practice of prophylactic removal of pathology-free impacted third molars should be discontinued”; specific attention must be drawn in patients with no compliance, who are therefore being exposed to the risk of undertaking general anesthesia unnecessarily.

Effective patient management is discussed in the context of the problem-oriented approach; indeed, early diagnosis, proper evaluation, and appropriate treatment are essential for developing an action plan.

A systematic approach to diagnosis and treatment planning involves clinical and radiographic examination that encompasses information regarding patients’ health status, age, and compliance.

Syndrome patients often present third molars with different crown shapes and sizes, root morphology, position and inclination, eruption paths, development periods, and relationships with contiguous anatomical structures.

Young patients revealed high incidence for caries and infection due to poor oral health education at home, high sugar consumption, and no strict compliance for daily brushing; this factor may lead to the premature loss of teeth [30]. The rationale of a removal of third molars is to monitor hygienic situation and avoid the extraction of tooth germs in case of missing teeth.

Surgical or nonsurgical treatment of third molars in syndrome/incompliant patients should be viewed with the perspective of the “daughter theory”. Considering the quality of life as the main goal of our job, is important to discuss with parents/caregivers about surgical and nonsurgical treatments alternatives, considering patients’ conditions and preferences.

## Figures and Tables

**Table 1:** Characteristics of the included studies.

Author (Year)	Study Type	Sample Size	Population Details	Male/Female% (Age ± SD)	Complications	Follow-Up (Months ± SD)
Chaparro-Avendaño et al. (2005)	Retrospective	173	Patients subjected to third molar surgical extraction in a Master University Course	33.1/66.9	Pain and swelling, infection, trismus, ecchymosis, and IAN and LN paresthesia	NR
				Group A (12–14 yr)		
				Group B (15–16 yr)		
				Group C (17–18 yr)		
Jasinevicius et al. (2008)	Recommendations	146	Questionnaires mailed to department chairpersons regarding recommendations about prophylactic third molar extraction	NR	NR	Not Applicable
				Adolescent (<21 yr)		
				Young Adult (21–35 yr)		
				Adult (>35 yr)		
Cunha-Cruz et al. (2014)	Prospective	797	Cohort of patients enrolled by dentists as part of the network named PRECEDENT (Northwest Practice-based REsearch Collaborative in Evidence-based DENTistry)	50.9/49.1	NR	19.7 ± 5.3
				>16 yr		
Cassetta et al. (2017)	Prospective	14	Patients subjected to third molar surgical extraction in a University Dental Clinic	50/50 (12.9 ± 0.5)	None	0.23 ± NR

IAN = Inferior Alveolar Nerve; LN = Lingual Nerve; NR = Not Reported.

**Table 2:** Review of author judgments on quality assessment for each included study.

Item	Chaparro-Avendaño et al.	Jasinevicius et al.	Cunha-Cruz et al.	Cassetta et al.
Clear stated aim	2	2	2	2
Inclusion of consecutive patients	1	0	1	1
Unbiased assessment of the study endpoint	2	2	2	2
Appropriate follow-up	0	0	2	1
Loss to follow-up less than 5%	2	1	1	2
Appropriate calculation of the study size	0	0	0	0
Adequate statistical analyses	2	2	2	2
TOTAL	9	7	10	10
Overall risk of bias	Medium	High	Medium	Medium

0 = Not reported, 1 = not adequately assessed, 2 = adequately assessed.

**Table 3:** Clinical recommendations for syndrome/incompliant patients.

	Early Removal	Late Removal
Indications	Morphologic abnormalities	Orthodontic considerations (dentoalveolar discrepancy, distalization of upper molars)
	Ekman–Westborg–Julin syndrome	
	Self-mutilating behavior or recurrent epilepsy	Patients with increased risk of tooth loss

## Supplementary Materials

The following are available online at https://www.mdpi.com/1010-660X/55/3/79/s1, Figure S1: PRISMA-P (Preferred Reporting Items for Systematic review and Meta-Analysis Protocols) 2015 checklist: recommended items to address in a systematic review protocol.
